# Hemostasis achieved with new self-assembling peptide hemostatic gel for a bleeding duodenal diverticulum

**DOI:** 10.1055/a-2655-4479

**Published:** 2025-08-08

**Authors:** Yu Ebisawa, Hideyuki Chiba, Akimichi Hayashi, Mikio Kobayashi, Jun Arimoto, Hiroki Kuwabara, Michiko Nakaoka

**Affiliations:** 1 74155 Department of Gastroenterology, Omori Red Cross Hospital, Ota-ku, Japan

Endoscopic hemostasis of parapapillary diverticular hemorrhage is challenging due to poor visualization caused by blood pooling and difficulty with endoscope manipulation. There have been reports of cases that required interventional radiology or surgical treatment. Recently, a new self-assembling peptide hemostatic gel (PuraStat; 3-D Matrix Ltd., Tokyo, Japan) has become available for treatment of gastrointestinal bleeding 1 2 . We report the first case of hemostasis achieved with PuraStat for a bleeding duodenal diverticulum.

A 75-year-old man receiving aspirin therapy presented to the emergency department with hematemesis. Upper gastrointestinal endoscopy revealed active hemorrhage in the diverticulum near the duodenal papilla ( Fig. 1 ). Persistent venous hemorrhage rapidly resulted in blood pooling within the parapapillary diverticulum ( Fig. 2 ). Electrocoagulation could not be performed due to the risk of perforation. We utilized 3 mL of PuraStat to fill the diverticulum for primary hemostasis ( Fig. 3 ). After a few minutes, the active bleeding subsided, permitting the identification of the exposed blood vessel ( Fig. 4 ). The vessel was secured using a reopenable clip ( Fig. 5 ), and complete hemostasis was achieved without complications ( Video 1 ).

**Fig. 1:**
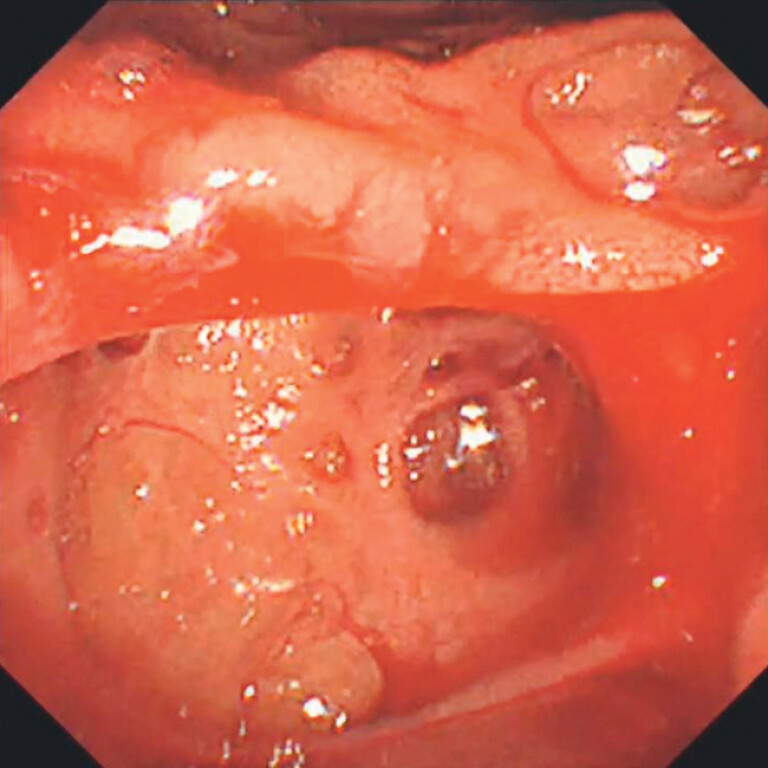
Suspected bleeding on the oral side (duodenoscope TJF-260V; Olympus, Tokyo, Japan).

**Fig. 2:**
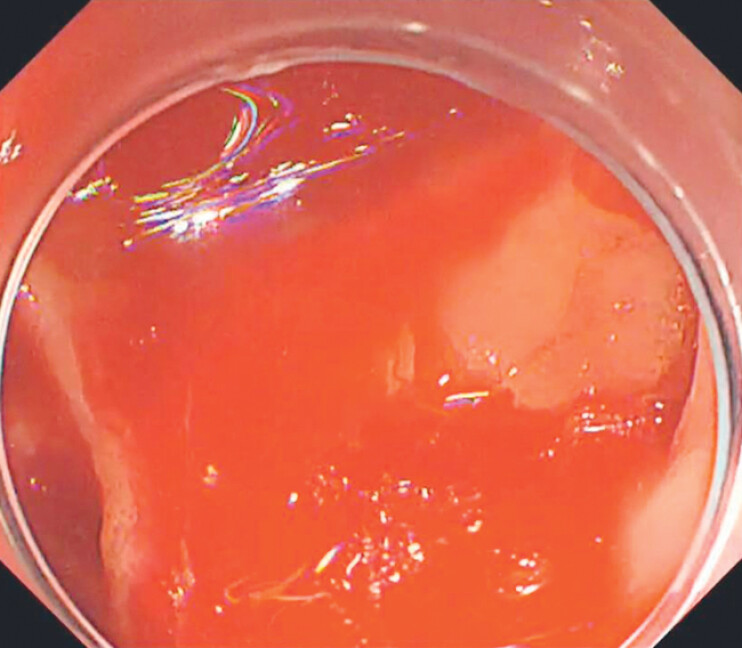
Limited visual field due to blood accumulating in the diverticulum (gastroscope GIF-H290T; Olympus, Tokyo, Japan).

**Fig. 3:**
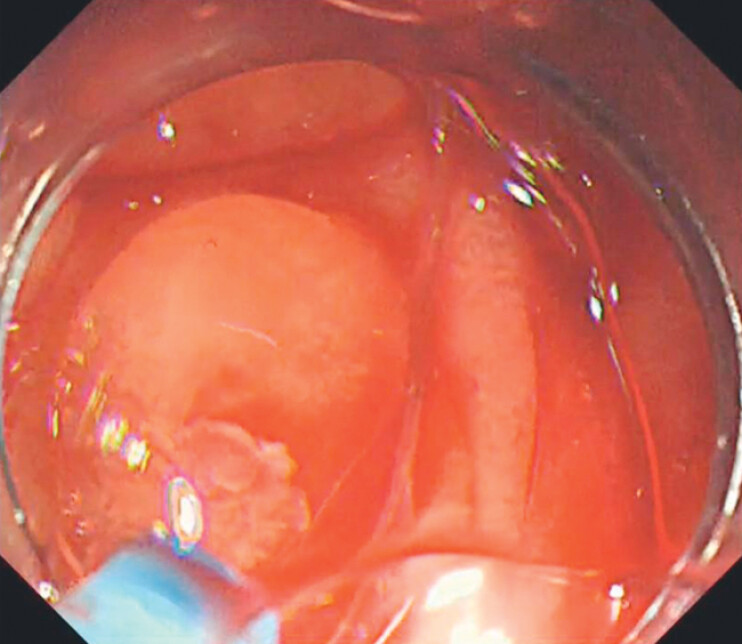
Primary hemostasis with PuraStat (3-D Matrix Ltd., Tokyo, Japan).

**Fig. 4:**
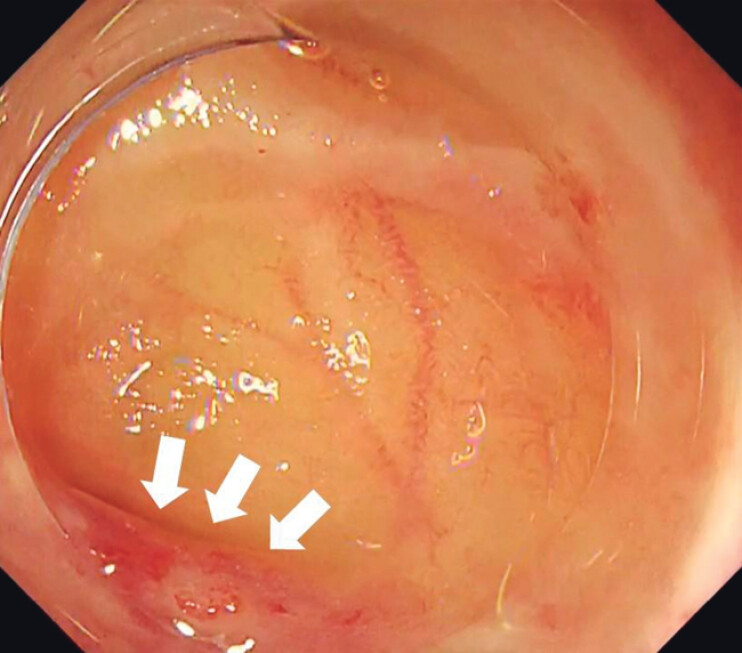
Primary hemostasis allowed a good visual field and vascular identification.

**Fig. 5:**
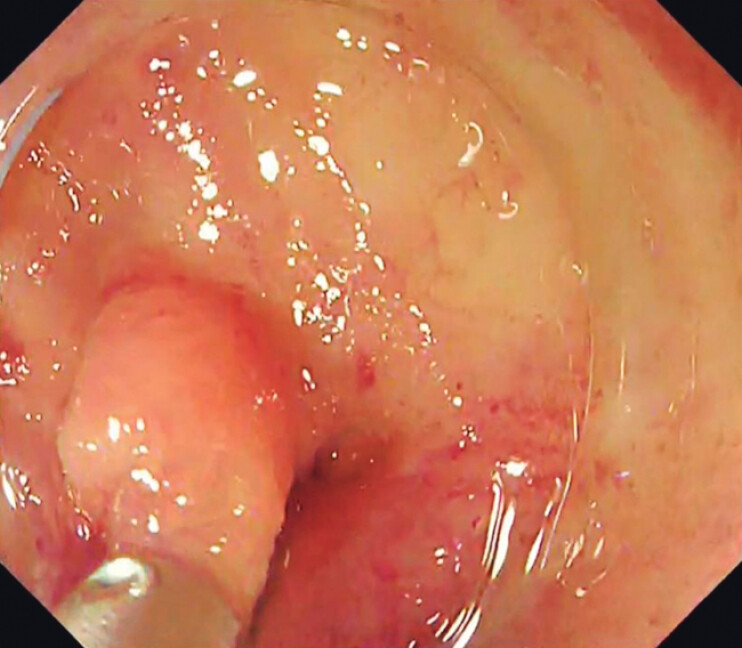
Hemostasis was achieved with a clip.

PuraStat (3-D Matrix Ltd., Tokyo, Japan) may offer a new strategy for managing duodenal diverticular hemorrhage.Video 1

PuraStat reduced the bleeding and ensured a clear visual field, resulting in endoscopic hemostasis. PuraStat was deemed beneficial as a potential hemostatic treatment for parapapillary diverticulum hemorrhage, especially in active bleeding where maintaining visual clarity is difficult. PuraStat should be considered in emergency endoscopic hemostasis of parapapillary diverticulum bleeding.

Endoscopy_UCTN_Code_TTT_1AO_2AD

## Notes

Endoscopy E-Videos https://eref.thieme.de/e-videos
*E-Videos* is an open access online section of the journal *Endoscopy* , reporting on interesting cases and new techniques in gastroenterological endoscopy. All papers include a high-quality video and are published with a Creative Commons CC-BY license. Endoscopy E-Videos qualify for HINARI discounts and waivers and eligibility is automatically checked during the submission process. We grant 100% waivers to articles whose corresponding authors are based in Group A countries and 50% waivers to those who are based in Group B countries as classified by Research4Life (see: https://www.research4life.org/access/eligibility/ ). This section has its own submission website at https://mc.manuscriptcentral.com/e-videos .
